# Identifying Causal Genes at the Multiple Sclerosis Associated Region 6q23 Using Capture Hi-C

**DOI:** 10.1371/journal.pone.0166923

**Published:** 2016-11-18

**Authors:** Paul Martin, Amanda McGovern, Jonathan Massey, Stefan Schoenfelder, Kate Duffus, Annie Yarwood, Anne Barton, Jane Worthington, Peter Fraser, Stephen Eyre, Gisela Orozco

**Affiliations:** 1 Arthritis Research UK Centre for Genetics and Genomics, Centre for Musculoskeletal Research, Division of Musculoskeletal and Dermatological Siences, Faculty of Biology, Medicine and Health, Manchester Academic Health Science Centre, The University of Manchester, Stopford Building, Oxford Road, Manchester, M13 9PT, United Kingdom; 2 Nuclear Dynamics Programme, The Babraham Institute, Cambridge CB22 3AT, United Kingdom; 3 NIHR Manchester Musculoskeletal BRU, Manchester Academic Health Sciences Centre, Central Manchester Foundation Trust, Manchester, United Kingdom; Westmead Millennium Institute for Medical Research, AUSTRALIA

## Abstract

**Background:**

The chromosomal region 6q23 has been found to be associated with multiple sclerosis (MS) predisposition through genome wide association studies (GWAS). There are four independent single nucleotide polymorphisms (SNPs) associated with MS in this region, which spans around 2.5 Mb. Most GWAS variants associated with complex traits, including these four MS associated SNPs, are non-coding and their function is currently unknown. However, GWAS variants have been found to be enriched in enhancers and there is evidence that they may be involved in transcriptional regulation of their distant target genes through long range chromatin looping.

**Aim:**

The aim of this work is to identify causal disease genes in the 6q23 locus by studying long range chromatin interactions, using the recently developed Capture Hi-C method in human T and B-cell lines. Interactions involving four independent associations unique to MS, tagged by rs11154801, rs17066096, rs7769192 and rs67297943 were analysed using Capture Hi-C Analysis of Genomic Organisation (CHiCAGO).

**Results:**

We found that the pattern of chromatin looping interactions in the MS 6q23 associated region is complex. Interactions cluster in two regions, the first involving the rs11154801 region and a second containing the rs17066096, rs7769192 and rs67297943 SNPs. Firstly, SNPs located within the *AHI1* gene, tagged by rs11154801, are correlated with expression of *AHI1 and* interact with its promoter. These SNPs also interact with other potential candidate genes such as *SGK1* and *BCLAF1*. Secondly, the rs17066096, rs7769192 and rs67297943 SNPs interact with each other and with immune-related genes such as *IL20RA*, *IL22RA2*, *IFNGR1* and *TNFAIP3*. Finally, the above-mentioned regions interact with each other and therefore, may co-regulate these target genes.

**Conclusion:**

These results suggest that the four 6q23 variants, independently associated with MS, are involved in the regulation of several genes, including immune genes. These findings could help understand mechanisms of disease and suggest potential novel therapeutic targets.

## Introduction

Genome wide association studies (GWAS) have been pivotal in identifying genetic associations with single nucleotide polymorphisms (SNPs) in many complex diseases, including multiple sclerosis (MS) [1–4]. MS is an inflammatory demyelinating disease of the central nervous system (CNS) and is a common cause of chronic neurological disability, showing moderate heritability (λ_s_ ~6.3) [5]. Similar to many autoimmune diseases, the major histocompatibility complex (MHC) represents the largest single genetic risk factor for MS, with multiple non-HLA loci, discovered in large international GWAS, contributing smaller individual effects to disease susceptibility. Due to the extensive overlap of genetic loci between multiple autoimmune diseases, the International Multiple Sclerosis Genetics Consortium (IMSGC) conducted a large study using the Illumina Immunochip genotyping array, identifying 48 new and validating 49 previously discovered non-MHC susceptibility variants for MS [6]. Among these variants, four mapped to the 6q23 region, which is also associated with other autoimmune diseases including rheumatoid arthritis (RA), systemic lupus erythematosus (SLE), celiac disease (CeD), type 1 diabetes (T1D), inflammatory bowel disease (IBD), psoriasis (Ps) and psoriatic arthritis (PsA), and containing several candidate genes, such as *TNFAIP3*, *AHI1* and *IL22RA2* [7–13].

**Fig 1:**
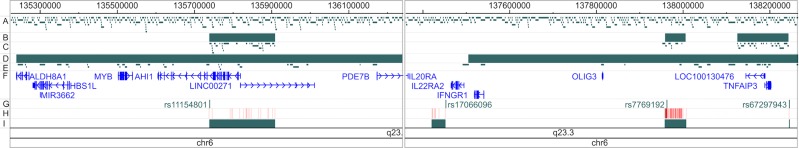
Overview of MS 6q23 Immunochip associated regions. Tracks are labelled as follows: A–HindIII restriction fragments; B–LD regions targeted in ‘region’ Capture Hi-C; C–HindIII restriction fragments targeted in ‘region’ Capture Hi-C; D–Gene regions targeted in ‘promoter’ Capture Hi-C; E–HindIII restriction fragments targeted in ‘promoter’ Capture Hi-C; F–RefSeq genes (packed for clarity); G–MS index SNPs; H–Density of MS LD SNPs (r^2^ ≥ 0.8) and I–MS LD regions. The genomic region chr6:136,238,000–137,360,000 has been omitted for clarity. All co-ordinates are based on GRCh37. Generated using the WashU EpiGenome Browser (http://epigenomegateway.wustl.edu/browser/).

The 6q23 locus, like many other GWAS loci, shows extensive overlap with many other autoimmune diseases and demonstrates a complex pattern of different associations attributable to different diseases. This sharing of associated loci led to the Immunochip array which contains three regions densely mapped and capturing four independent associations with MS (**Table 1 and Fig 1**) in the 6q23 region. The first, tagged by rs11154801, is located within an intron of the *AHI1* gene required for both cerebellar and cortical development. The second region, tagged by rs17066096, is an intergenic region 87kb 5’ of *IL20RA* and 12kb 3’ of *IL22RA2*. The third region covers 430kb, encompassing a *PTPN11* pseudogene (*RP11-95M15*.*2*), *TNFAIP3* and several lncRNAs and contains two independent associations (rs7769192 & rs67297943). Interestingly, while other SNP associations are shared between autoimmune diseases, the MS associated SNPs are unique to MS alone (**S1 Data**). As such, these MS associated SNPs could offer an insight into the mechanisms affecting MS at this locus.

**Table 1:** MS 6q23 Immunochip associated regions.

Region Co-ordinates (GRCh37)			Index SNP	Reported Gene	MAF	P	OR
Chr.	Start	End					
6	134946991	135498875	rs11154801	*AHI1*	0.37	1.80 x 10^−20^	1.12
6	136685539	136875216	rs17066096	*IL22RA2*	0.23	1.60 x 10^−23^	1.14
6	137231416	137660037	rs7769192^a^	*-*	0.45	3.30 x 10^−09^	1.08
			rs67297943	*TNFAIP3*	0.22	5.50 x 10^−13^	1.11

All P values are from the joint analysis by Beecham et al.[6]. Chr., chromosome; MAF, minor allele frequency; OR, odds ratio.

a, P values and ORs shown after conditioning on rs67297943.

However, due to the design of GWAS, these lead genetic associations do not necessarily represent the causal variant but instead a number of variants in strong linkage disequilibrium with them. In addition, associated SNPs have generally been annotated to the closest, most biologically plausible gene. Evidence suggests that GWAS discovered SNPs in general, including these associations within 6q23, are enriched in cell-type specific enhancer regions [14,15] which can regulate gene expression. Additionally, an individual’s genotype can influence this expression (expression quantitative trait loci (eQTL)), potentially leading to disease. It has been shown that enhancers can regulate genes located some distance away through long-range chromatin interactions [16]. Therefore, confidently assigning causal SNPs, genes and cell types to these and other GWAS signals remains a major challenge. Potential long-range interactions have previously been prohibitive to investigate as methods, such as 3C and Hi-C, required interacting regions to be considered *a priori* or, lacked throughput and resolution. Capture Hi-C was developed to overcome these limitations by enriching a Hi-C library using RNA baits designed to specific restriction fragments. This approach reduces library complexity, increases power and subsequently allows the identification of statistically significant chromatin interactions at a restriction fragment resolution (~4kb). As part of a large study investigating the interactions with associated regions in four autoimmune diseases [17], several sites within the 6q23 region were targeted, including associated regions and promoters of nearby genes (**Table 1** and **Fig 1**). Our Capture Hi-C data represents a unique opportunity to explore this region for MS and offer an insight into the mechanisms specifically affecting MS at this locus and how they compare with other autoimmune diseases. The aim of this study was to use this chromatin interaction experiment to explore the unique genetic associations with MS in the 6q23 region to identify possible target causal genes whose expression could be perturbed in at risk individuals. The ultimate goal is to help translate GWAS findings into clinical benefit, as the identification of causal genes can pinpoint biological mechanisms altered in disease and suggest potential therapeutic targets or drug repositioning.

We demonstrate that the MS associated region 6q23 presents numerous, complex chromatin looping interactions clustered in two regions. The first contains SNPs located within the *AHI1* gene, tagged by rs11154801, and correlated with expression, which interact with the *AHI1* promoter thereby supporting the gene candidature of *AHI1*. Interestingly, these SNPs also interact with other potential candidate genes such as *SGK1* and *BCLAF1*, suggesting they may regulate multiple loci. The second region encompasses the rs17066096, rs7769192 and rs67297943 associated regions and interact with each other and with immune-related genes, such as *IL20RA*, *IL22RA2*, *IFNGR1* and *TNFAIP3*. Additionally, these regions interact with each other and therefore, may co-regulate these target genes.

## Materials and Methods

### MS SNP Associations & Regions

All MS SNP associations in the 6q23 region were taken from the IMSGC Immunochip study [6]. All SNPs in linkage disequilibrium (LD) (r2≥0.8) with each lead Immunochip MS SNP were identified using European samples from the 1000 Genomes Phase 3 release. Associated regions for each lead association were defined by the two terminal SNPs in LD.

### Cell culture

B-lymphoblastoid cell lines (LCL) were obtained directly from Coriell Institute for Medical Research (catalogue number GM12878). Cells were grown in vented 25cm^2^ cell culture flasks containing 10-20mls of Roswell Park Memorial Institute (RPMI)-1640 + 2mM L-glutamine culture medium, supplemented with 15% foetal bovine serum (FBS). Flasks were incubated upright at 37°C/5% CO^2^. Cultures were regularly monitored to maintain a cell density between 2×10^5^–5×10^5^ viable cells/ml. Cells were split every 2 days into fresh medium until they reached a maximum density of 1×10^6^ cells/ml.

Jurkat E6.1 human leukaemic T-lymphoblast cells were obtained directly from LGC Standards (catalogue number ATCC^®^ TIB-152^™^). Cells were grown in vented 25cm^2^ cell culture flasks containing 10-20mls of RPMI-1640 + 2mM L-glutamine, supplemented with 10% FBS. Flasks were incubated upright at 37°C/5% CO_2_ and the cultures regularly monitored to maintain a cell density between 3×10^5^–9×10^5^ viable cells/ml.

These cell lines are not listed in the in the database of commonly misidentified cell lines maintained by ICLAC, were authenticated using STR analysis and were tested for mycoplasma contamination (MycoSEQ® Mycoplasma Detection System, 4460625, Life Technologies).

### Capture Hi-C

Capture Hi-C data was produced as part of a larger study targeting all regions associated with four autoimmune diseases (RA, JIA, PsA and T1D) and separately, all promoters within these regions [17]. Briefly, all promoters within 1Mb of associated SNPs were selected and RNA baits were designed to the ends of all fragments within 500bp of the transcription start sites. Separately, associated regions were defined by SNPs in LD (r^2^≥0.8) and all restriction fragments not selected for the promoter capture experiment were targeted. Experiments were performed using human T-cell (Jurkat) and B-cell (GM12878) lines. Capture Hi-C libraries were sequenced using 75bp paired-end reads on an Illumina HiSeq 2500. Resulting reads were mapped to restriction fragments and filtered using the Hi-C User Pipeline (HICUP http://www.bioinformatics.babraham.ac.uk/projects/hicup). Chromatin interactions were analysed using CHiCAGO (Capture Hi-C Analysis Of Genomic Organisation [18], http://regulatorygenomicsgroup.org/chicago), a publicly available, open-source, bespoke statistical model for detecting significant interactions in Capture Hi-C data at a single restriction fragment resolution. Further filtering was carried out using the BEDTools v2.21.0 pairtobed command to identify significant interactions involving the MS associated regions.

Chromatin interactions identified in the Capture Hi-C data were further validated against dense Hi-C data generated by Rao *et al*. [19] in GM12878 cells. No data was available for the Jurkat T-cell line. Raw contact matrices and normalisation matrices for GM12878 cells at 5kb resolution were obtained from GEO accession GSE63525. Observed and expected contact matrices were normalised using the Knight and Ruiz normalisation matrices as described in the accompanying documentation. Observed/expected (O/E) values were calculated and further filtered by O/E ≥5 and normalised read count ≥ 5. BEDTOOLS was used to obtain the overlap of interactions observed in our data and the Rao *et al*. [19] data.

### Expression quantitative trait loci (eQTLs) analysis

Publicly available datasets from Westra, *et al*. [20], the GEUVADIS analysis (http://www.ebi.ac.uk/arrayexpress/files/E-GEUV-1/analysis_results/) and Raj *et al*. [21] were queried directly. Additional datasets were also queried through HaploReg [22]. Two whole-genome gene expression datasets were also available in-house: CD4+ and CD8+ T-cells from 21 healthy individuals of the National Repository of Healthy Volunteers (NRHV), The University of Manchester. Written informed consent was obtained from all subjects. Ethical approval was obtained from North West Centre for Research Ethics Committee (REC: 99/8/84). Samples were all of Caucasian ancestry with a median age of 50.5 years (26–82 years) and comprised of 8 males and 13 females. mRNA was isolated from sorted cell subsets, quality and concentration assessed using the Agilent Bioanalyzer and Nanodrop, before cDNA/cRNA conversion using Illumina TotalPrep RNA Amplification Kits. 750ng of cRNA was hybridised to HumanHT-12 v4 Expression BeadChip arrays according to the manufacturer’s protocol before being scanned on the Illumina iScan system. Raw expression data were exported from Illumina GenomeStudio and analysed using the R Bioconductor package ‘limma’ 81. Briefly, the neqc function was used for log2 transformation of the data, background correction and quantile normalisation using control probes. Principal Component Analysis was used to detect batch effects. The cDNA/cRNA conversion produced the largest batch effect in both cohorts and was corrected using ComBat (in R Bioconductor package sva) (http://bioconductor.org/packages/release/bioc/html/sva.html). Genome-wide genotype data was generated using the Illumina HumanCoreExome BeadChip kit. Genotype data was aligned to the 1000 genomes reference strand, pre-phased using SHAPEIT2 (v2.r727), before imputation using IMPUTE2 (v2.3.0) with the 1000 genome reference panel Phase 1. Imputed data was hard-called to genotypes using an INFO score cut-off of 0.8 and posterior probability of 0.9. The effect of the SNPs on gene expression was analysed using MatrixEQTL (v 2.1.0) (http://www.bios.unc.edu/research/genomic_software/Matrix_eQTL/) with an additive linear model. Only SNPs within 4Mb of a gene expression probe were considered to be cis-eQTL.

### Bioinformatics Refinement of SNPs

SNPs were annotated using data from HaploReg v4.1 [22] and RegulomeDB v1.1 [23] for each LD SNP and combined with our Capture Hi-C data. SNPs attaining a RegulomeDB score of ≥5 and showing evidence of chromatin interactions in either cell type were selected as potentially causal and merit further investigation.

## Results and Discussion

### Capture Hi-C

Chromatin interactions at the 6q23 locus were analysed as part of a larger study that included all known risk loci for RA, juvenile idiopathic arthritis (JIA), PsA and T1D. We performed two different Capture Hi-C experiments: firstly, the Region Capture targeted the LD regions (r^2^>0.8) for all SNPs associated with each disease (**Fig 1B**); secondly, the Promoter Capture targeted all known gene promoters overlapping a region 500kb upstream and downstream of the lead disease associated SNP (**Fig 1D**). Capture Hi-C libraries were generated for two cell lines: GM12878, a B-lymphoblastoid cell line, and Jurkat, a CD4+ T-lymphoblastoid cell line.

**Fig 2:**
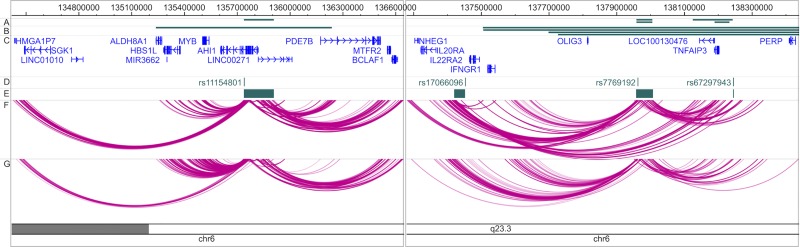
Overview of MS 6q23 interactions. Tracks are labelled as follows: A–LD regions targeted in ‘region’ Capture Hi-C; B–Gene regions targeted in ‘promoter’ Capture Hi-C; C–RefSeq genes (packed for clarity); D–MS index SNPs; E–MS LD regions; F–Interactions observed in the GM12878 B-cell line and G–Interactions observed in the Jurkat T-cell line. Promoter and region Capture Hi-C experiments have been merged for clarity. The genomic region chr6:136,650,000–137,280,000 has been omitted for clarity. All co-ordinates are based on GRCh37. Generated using the WashU EpiGenome Browser (http://epigenomegateway.wustl.edu/browser/).

Our Capture Hi-C experiments revealed that the 6q23 region presents a complex pattern of chromosomal interactions, highlighting both new and previously implicated genes for disease risk. Overall, 827 unique interactions involving MS 6q23 associated regions were observed across both cell lines and both capture experiments (promoter & region) (**Fig 2** and **S1 Fig**). Each cell line demonstrated similar interaction patterns in both capture experiments. Encouragingly, there was a high degree of support from previously published Hi-C data obtained in GM12878 cells [19], with between 81% and 90% of interactions identified through our Capture Hi-C also being seen at an observed/expected ratio of ≥5 in previously published data.

The numerous chromosomal interactions detected appeared to cluster in two genomic locations, involving the rs11154801 region and a region containing the other three independent MS associations: rs17066096, rs7769192 and rs67297943 (**Fig 2**).

The rs11154801 LD block spans 170.4kb and contains several enhancers overlapping SNPs in strong LD with the lead association. It demonstrates a complex array of several long-range (>100kb) as well as shorter (<100kb) chromatin interactions (**Fig 2** and **S2 Fig**) in both cell lines. Shorter internal chromatin interactions include ones with restriction fragments flanking the *AHI1* gene (previously assigned as the candidate gene to this variant) promoter, and SNPs in LD with rs11154801. We show how these SNPs, within the introns of *AHI1*, interact with the promoter region thereby supporting the *AHI1* hypothesis in both cell lines. Mutations in *AHI1* have been shown to cause Joubert syndrome [24], an autosomal recessive neurological condition causing symptoms including neonatal breathing abnormalities and mental retardation. Furthermore, it has been suggested that *AHI1* is required for both cerebellar and cortical development in humans and is expressed in the brain [25].

However, this locus may be more complex than previously thought, as long-range chromatin interactions, although more numerous in B-cells, were observed between the enhancer region and other compelling candidates such as *SGK1* and *BCLAF1* in both cell lines. The interaction with *SGK1* represents a >1.2Mb interaction and *Sgk1* knockout mice have been shown to have a reduced incidence of disease severity in experimental autoimmune encephalomyelitis (EAE), a mouse model of MS [26]. Other long-range interactions include those to the *MYC* and *PDE7B* gene regions.

Finally, rs11154801 also showed an interaction in both B and T-cells with a region encompassing the promoters of *BCLAF1*, *MTFR2*, an antisense gene (*RP13-143G15*.*4*) overlapping *PDE7B* and a lincRNA (RP3-406A7.7). The *BCLAF1* gene encodes a transcriptional repressor which interacts with BCL2-family proteins, is expressed in the brain and overexpression can lead to cell apoptosis [27]. In lymphocytes, dysregulation of BCL2-family proteins has been shown to lead to a reduction of pro-apoptotic BCL-2 members and survival of T-cells in MS [28]. Additionally, *BCLAF1* has also been shown to be crucial in the homeostasis of T- and B-cell lineages and proliferation of T-cells [29]. These interactions suggest that the associations at this locus may have different or additional effects on disease than just the previously assigned *AHI1* gene.

The second cluster of chromatin interactions involve the remaining MS associated SNPs in the 6q23 region, rs17066096, rs7769192 and rs67297943. Our Capture Hi-C results showed that these three SNPs interact with each other and also with several genes with immune function such as *IL20RA*, *IL22RA2*, *IFNGR1* and *TNFAIP3*, suggesting that these variants may be involved in the regulation of common immune pathways (**Fig 2**).

The first two interacting regions in this cluster, identified by the promoter Capture Hi-C experiment and tagged by rs17066096, were only observed in B-cells and involved the *IL22RA2* and *IFNGR1* gene promoters, almost 71kb and 113kb away, respectively. The restriction fragment overlapping the rs17066096 LD block shows evidence of enhancer activity, as predicted by ChromHMM, and the furthest 5’ SNP in LD with the index SNP is located within this enhancer.

The region tagged by rs7769192 spans 50kb and contains 72 SNPs in LD, 21 of which are perfectly correlated with the lead SNP. It shows evidence of multiple enhancers and in addition to the previously mentioned interaction with the rs17066096 region, interacts with five other regions, in both cell lines. The first of these is located >500kb 5’ of rs7769192 and contains the *IL20RA* gene. The next two regions, located over 400kb away, are shared with the rs17066096 region and contain the *IL22RA2* and *IFNGR1* genes, providing further evidence of the interplay between the two associated regions. The product of the *IFNGR1* gene is a subunit of the interferon gamma (IFN-γ) receptor whose ligand, IFN-γ, is important in adaptive immunity and has been linked to many different autoimmune diseases [30]. The *IL20RA* and *IL22RA2* genes both encode receptors for members of the IL-20 sub-family of cytokines and both exhibit a pro-inflammatory effect [31]. Additionally, anti-IL20 therapy has recently been shown to be effective in the treatment of RA and psoriasis [32,33]. Although anti-IL20 therapy was not developed as a result of Capture Hi-C, the discovery of interactions between these genes and autoimmune disease associations, demonstrates the power of this technique to inform drug discovery or repositioning. Further evidence for anti-IL20 therapy comes from the interaction with *IL22RA2*. *IL22RA2* encodes a soluble receptor which binds to and inhibits IL-22, a cytokine which can stimulate pro-inflammatory epithelial defence mechanisms [34], preventing the interaction with its cell surface receptor. This evidence suggests that blocking the IL-20 pathway may be effective in the treatment of MS and other autoimmune diseases.

The fourth region is located 178kb 3’ of the associated region and contains multiple promoters of the *TNFAIP3* gene as well as a non-coding processed transcript (RP11-356I2.4) of unknown function. The role of *TNFAIP3* in autoimmunity is well established and the gene product A20 is a protein that is induced by tumour necrosis factor (TNF) and inhibits NFκB activation and TNF-mediated apoptosis [35]. This locus within the 6q23 region is one of the most important autoimmunity risk loci, as it contains multiple SNPs strongly associated with many autoimmune diseases, including MS, RA, SLE, CeD, IBD, psoriasis and PsA, among others. Variants associated with most autoimmune diseases map to the *TNFAIP3* gene or its vicinity, including the MS SNPs rs17066096, rs7769192 and rs67297943.

The rs67297943 SNP is located within a predicted enhancer element in B-cells and also in a 48.8kb region showing multiple enhancer marks in both B and T-cells. Furthermore, the adjacent restriction fragment to rs67297943 interacts with the *IL20RA* promoter region only in B-cells in this experiment.

Our Capture Hi-C results suggest that rs17066096, rs7769192 and rs67297943 physically interact with several immune genes with pro-inflammatory roles, such as *IL20RA*, *IL22RA2*, *IFNGR1* and *TNFAIP3*, indicating that they may be involved in the inflammatory processes that typically occurs in autoimmunity. Conversely, rs11154801, which is exclusively associated to MS and not other autoimmune diseases, interacts with genes with neurological function, like *AHI1*, *SGK1* and *BCLAF1*. Intriguingly, these two separate regions, over 2.3Mb apart, interact with each other in T-cells but not B-cells (**S1 Fig**), suggesting that these pathways may converge to give rise to disease-specific MS mechanisms in a stimulus and cell type specific manner. In this regard, it has been previously shown that there is a correlation between chromatin interactions and gene co-expression [36–39] and it has been hypothesised that multiple co-regulated genes can interact and share regulatory elements at specialised ‘transcription factories‘ [16]. Our data possibly supports this idea and suggests a possible co-regulation of genes in this region in MS.

It could be argued that the differences observed between cell types for the rs1154801 region and the rs17066096 region could also be attributable to genotype differences in the cell lines (**Table 2**). While the overall pattern of chromatin interactions is similar for the rs11154801, rs17066096 and MYB regions, the intensity of interactions observed does vary between cell lines and could be due to carriage of risk alleles in the B-cell line, absent in the T-cell line. It is therefore important to validate the chromatin interactions in a genotype specific manner.

**Table 2:** Associated SNP genotypes for GM12878 (B) and Jurkat (T) cell lines.

SNP	Risk Allele	GM12878		Jurkat	
		Genotype	Number of risk alleles	Genotype	Number of risk alleles
rs11154801	A	CA	1	CC	0
rs17066096	G	AA	0	AG	1
rs7769192	A	AA	2	unknown*	
rs67297943	C	TC	1	unknown*	

*Neither directly genotyped nor suitable proxies available on array

### Expression Quantitative Trait Loci (eQTLs)

Public databases were interrogated for evidence of eQTLs for MS associated SNPs in the 6q23 region (rs11154801, rs17066096, rs7769192 and rs67297943) and all SNPs in LD (r2>0.8) with them. The SNPs within the intergenic region 5’ of the *AHI1* gene, tagged by rs11154801 and interacting with the *AHI1* gene promoter, are correlated with *AHI1* mRNA expression in multiple tissues, including brain, nerve and whole blood. This further supports that *AHI1* is one of the causal genes within the 6q23 region.

Although many of the interactions detected in the Capture Hi-C experiment are between regions which show enhancer activity and regions which show active transcription, no eQTL evidence from public databases was observed for any of the other MS associated SNPs investigated, other than rs11154801. This could be due to the distance cut-offs used to define *cis* effects or the selection of the correct cell type and stimulatory conditions, as eQTLs are known to be highly cell type and stimulus specific.

**Fig 3:**
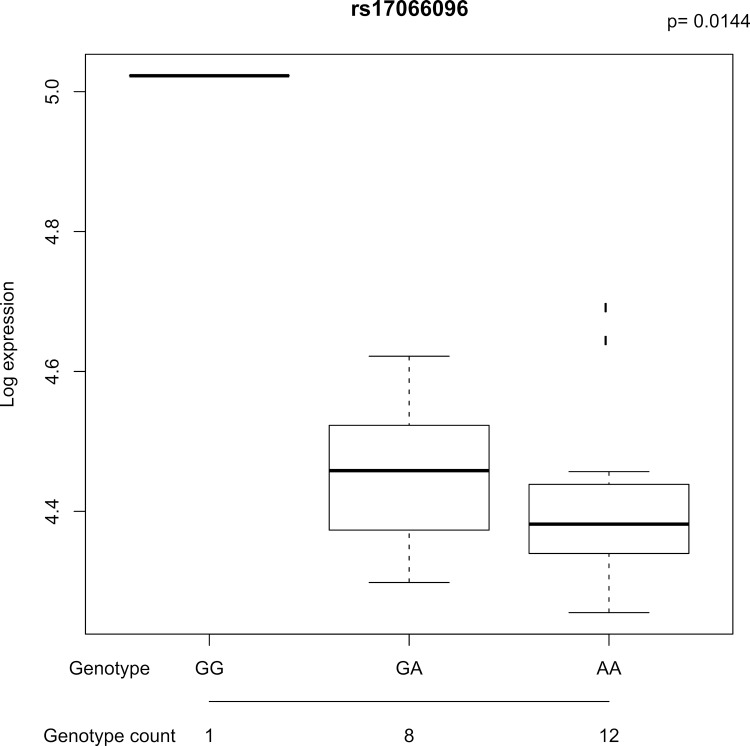
Increased expression of *IL20RA* in primary CD8+ T-cells from 21 healthy individuals carrying the G risk allele of rs17066096, *P* = 0.01. The three different genotypes for the SNPs are displayed on the X axis and gene expression levels on the Y axis. Error bars indicate standard deviation.

However, using in-house eQTL data on CD4+ and CD8+ primary T-cells from healthy donors, rs17066096 was shown to be correlated with expression of *IL20RA* in CD8+ T-cells (*P* = 0.01, **Fig 3**). Although the design of the Capture Hi-C experiment did not allow for the testing of chromatin interactions between the rs17066096 region and *IL20RA*, this eQTL suggests *IL20RA* could be important in the pathogenesis of MS in CD8+ T-cells, which have previously been shown to contribute to disease [40]. This data also confirmed the eQTL between rs11154801 and *AHI1* in both CD4+ and CD8+ T-cells (P = 2.0x10^-4^ and 0.02 respectively). Full eQTL results for all MS SNPs are presented in **S2 Data**. No other eQTLs were identified between MS SNPs and genes showing chromatin interactions, this may again be due to the selection of the correct cell type and stimulatory conditions.

### Bioinformatics Refinement of SNPs

By utilising publically available data on regulatory elements obtained through HaploReg and RegulomeDB and augmenting with our Capture Hi-C data, we were able to refine large numbers of potential causal SNPs for three out of the four MS associated regions, by strong evidence of being in both a relevant cell-type enhancer region and interacting with a gene promoter (**Table 3** and **S3 Data**). For the rs11154801 region, 6 SNPs were identified out of 19 potential candidates; for the rs17066096, 3 SNPs were selected from a total of 7 in LD with the index association; and finally, for the rs7769192 region, we refined the potential candidates to 4 SNPs out of 72 in LD with rs7769192. No SNPs were found in LD with rs67297943 and although this SNP shows enhancer marks in 6 tissues, no interactions were identified with this region and as such further refinement was not possible. It will be imperative to follow-up putative SNPs and genes with functional assays and demonstrate their contribution to disease in relevant cell types in a biological context using genome editing techniques.

**Table 3:** Refined MS SNPs based on bioinformatics and Capture Hi-C data.

LD SNP	Index SNP	r^2^	RegulomeDB Score	Promoter histone marks	Enhancer histone marks	DNAse	Proteins bound	Motifs changed	GRASP QTL hits	Selected eQTL hits	GENCODE genes	Gene Interactions*
rs11154801	rs11154801	1	1d	BLD	BLD	BLD		Nkx2	20 hits	46 hits	AHI1	*AHI1*, *ALDH8A1*,*HBS1L*, *MYB*
rs7759971	rs11154801	0.99	1f	BLD	BLD, STRM, SKIN				1 hit	46 hits	AHI1	*ALDH8A1*, *HBS1L*, *MYB*, *SGK1*
rs13197384	rs11154801	0.92	1a	24 tissues		53 tissues	42 bound proteins	13 altered motifs	1 hit	44 hits	AHI1	*BCLAF1*, *HBS1L*, *HMGB1P17*, *MTFR2*, *PDE7B*
rs7750586^†^	rs11154801	0.98	1f	GI, BLD	10 tissues	22 tissues	CTCF, RAD21, SMC3		2 hits	53 hits	LINC00271	
rs9399148	rs11154801	0.95	1f		8 tissues	LNG, GI, BLD	KAP1	GR	2 hits	52 hits	LINC00271	*MTFR2*, *PDE7B*
rs5880258	rs11154801	0.95			4 tissues	MUS, MUS		Pbx-1		21 hits	LINC00271	*MTFR2*, *PDE7B*
rs1322553	rs17066096	0.96	5	BLD	IPSC, BLD	BLD		4 altered motifs			44kb 3' of *IL22RA2*	*IFNGR1*, *IL22RA2*, *TNFAIP3*
rs17066063	rs17066096	0.96	3a	BLD	BRST, BLD, SKIN	7 tissues	6 bound proteins	Pou2f2, TCF4	1 hit		41kb 3' of *IL22RA2*	*IFNGR1*, *IL22RA2*, *TNFAIP3*
rs12214014	rs17066096	0.97	5		BLD			GR			36kb 3' of *IL22RA2*	*IL22RA2*, *TNFAIP3*
rs9321623	rs7769192	0.97	4	FAT, SKIN, MUS	15 tissues	7 tissues		Rad21			36kb 5' of RP11-95M15.1	*IL20RA*, *IL22RA2*
rs7769192	rs7769192	1	4		6 tissues	18 tissues	CTCF	HNF1, Irf, Pax-6			32kb 5' of RP11-95M15.1	*IFNGR1*, *IL20RA*, *IL22RA2*
rs11758213^‡^	rs7769192	1	2a		GI, BLD, LIV	39 tissues	CTCF, RAD21, SMC3	4 altered motifs			12kb 5' of RP11-95M15.1	
rs10607561	rs7769192	0.93	3a			BLD	NFKB, SETDB1	Foxp1, RREB-1			4.2kb 3' of RP11-95M15.1	*TNFAIP3*

r^2^ has been rounded to 2 decimal places.

*—Only protein coding genes are shown for clarity.

^†^rs7750586 showed strong evidence of regulatory activity but was not involved in any interactions.

^‡^rs11758123 only interacts with lincRNAs. BLD, blood; BRST, breast; FAT, adipose tissue; GI, smooth muscle; IPSC, induced pluripotent stem cells; LIV, liver; LNG, lung; MUS, muscle; SKIN, skin; STRM, stromal connective.

## Conclusion

In conclusion, our work has strengthened the case for the *AHI1* gene candidate but also identified other potential MS gene targets, such as *SGK1*, *BCLAF1 IL20RA*, *IL22RA2*, *IFNGR1* and *TNFAIP3*. Additionally, we have shown a possible co-regulation of MS GWAS associations in the 6q23 region, which could help elucidate the pathogenesis of MS as well as other autoimmune diseases. These targets require further functional investigation which has been informed by the bioinformatics analysis. While the MS associations show evidence of interacting with other genes with no obvious role in MS pathogenesis, it is likely that they share regulatory elements within this region. It is however important to investigate these interactions, in addition to ones highlighted in this analysis to fully explore disease pathogenesis.

Whilst the interactions identified require further independent validation, the unique experimental design using complementary capture baits (region and promoter captures) provides robust evidence of chromatin interactions. Additionally, validation with chromatin interactions identified by Rao *et al*. [19] further add to the confidence of the observed interactions. While Capture Hi-C offers much greater resolution for chromatin interactions than Hi-C, observed interactions are still limited by the restriction enzyme used and do not pinpoint the interactions to specific enhancers. As such further work will be necessary to confirm causal enhancers and how they affect gene regulation. The use of cell lines is a limitation of the study but the experimental requirement of high cell numbers for Capture Hi-C makes the use of primary cells challenging. However, it is essential that further experiments are performed in primary cells to fully elucidate how chromatin interactions can effect gene regulation in MS. Despite these limitations of Capture Hi-C, it is clear that this technique is a powerful approach to link genes to their regulatory elements and this work has identified several candidate causal genes for MS. Additionally it has been proposed that by using genetic evidence to select drug targets, it could double the success rate in clinical development [41]. Since Capture Hi-C has the potential to identify causal genes for genetic associations, it provides a way to enhance this further. This is exemplified by the identification of chromatin interactions between MS associations and the *IL20RA* and *IL22RA2* genes, showing a potential use of anti-IL20 therapy in MS, and highlights the potential of Capture Hi-C to provide novel therapeutic targets or drug repositioning to improve patient outcome.

## Supporting Information

S1 Data LD between MS associated SNPs and other disease associated SNPs in the 6q23 region. Disease abbreviations are as follows: CEL–Coeliac disease; CRO–Crohn’s disease; MS–Multiple sclerosis; PBC–Primary biliary cirrhosis; PSO–Psoriasis; RA–Rheumatoid arthritis; SLE–Systemic lupus erythematosus; T1D –Type 1 diabetes; UC–Ulcerative colitis; SJO—Sjogren Syndrome.
(XLSX)

S2 Data NRHV CD4 and CD8 eQTLs for MS SNPs within the 6q23 region. (XLSX)

S3 Data Full table of bioinformatics and Capture Hi-C data analysis. Refined SNPs are highlighted in green.
(XLSX)

S1 Fig Interactions within the rs11154801 LD region. Tracks are labelled as follows: A–LD regions targeted in ‘region’ Capture Hi-C; B–Gene regions targeted in ‘promoter’ Capture Hi-C; C–GENCODE Genes V17; D–MS index SNPs; E–MS LD regions; F–Interactions observed in the GM12878 B-cell line and G–Interactions observed in the Jurkat T-cell line. All co-ordinates are based on GRCh37.
(TIF)

S2 Fig Full overview of MS 6q23 Immunochip associated regions. Tracks are labelled as follows: A–LD regions targeted in ‘region’ Capture Hi-C; B–Gene regions targeted in ‘promoter’ Capture Hi-C; C–RefSeq genes (packed for clarity); D–MS index SNPs; E–MS LD regions; F–Interactions observed in the GM12878 B-cell line and G–Interactions observed in the Jurkat T-cell line. All co-ordinates are based on GRCh37.
(TIF)
