# Supplementary figures and images for: Identifying Causal Genes at the Multiple Sclerosis Associated Region 6q23 Using Capture Hi-C
Source: PLoS One. 2016 Nov 18;11(11):e0166923. doi: 10.1371/journal.pone.0166923 (PMC5115837; doi:10.1371/journal.pone.0166923)

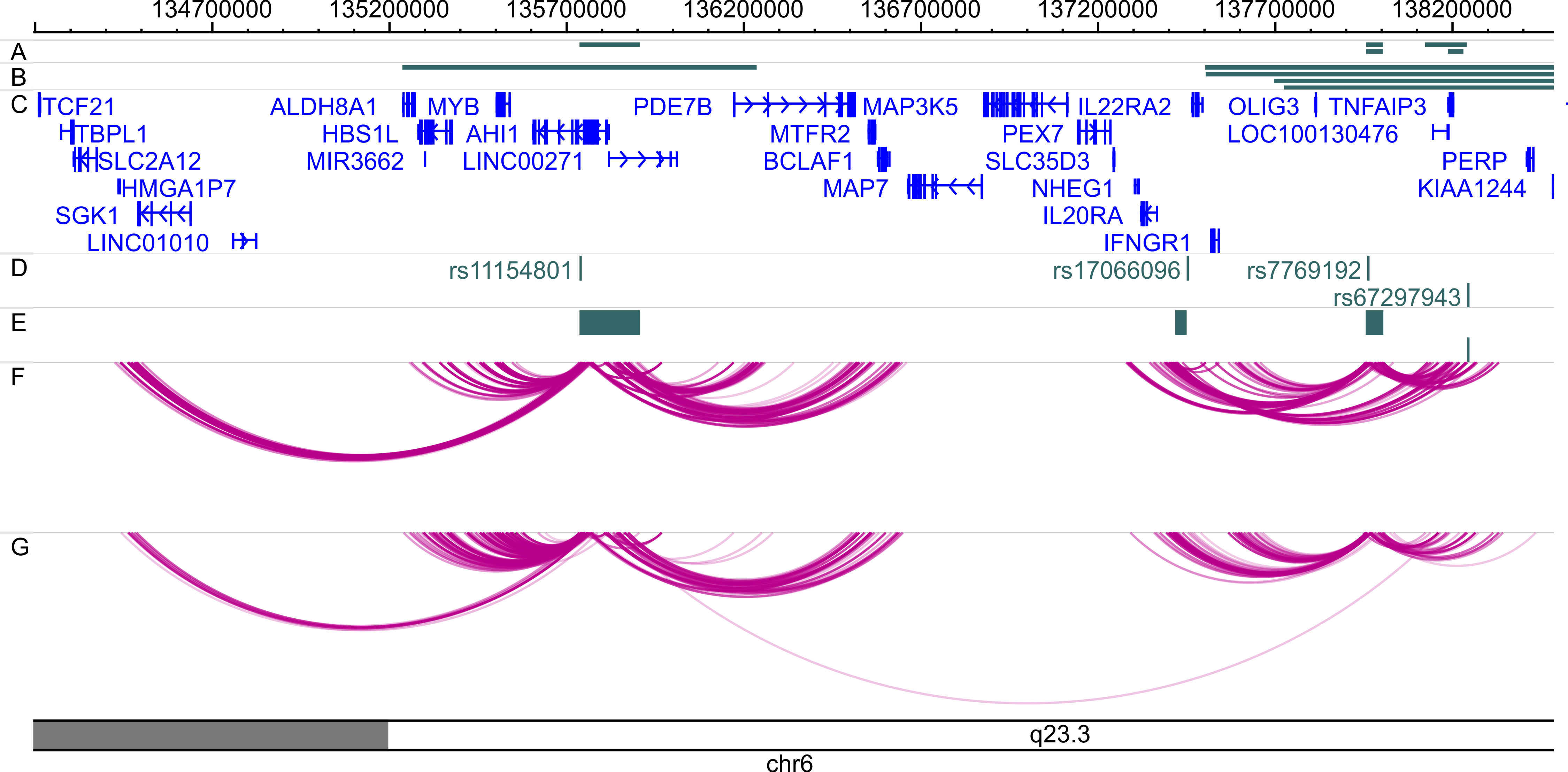

Supplement: S1 Fig — Tracks are labelled as follows: A–LD regions targeted in ‘region’ Capture Hi-C; B–Gene regions targeted in ‘promoter’ Capture Hi-C; C–GENCODE Genes V17; D–MS index SNPs; E–MS LD regions; F–Interactions observed in the GM12878 B-cell line and G–Interactions observed in the Jurkat T-cell line. All co-ordinates are based on GRCh37. (TIF) [file pone.0166923.s004.tif]

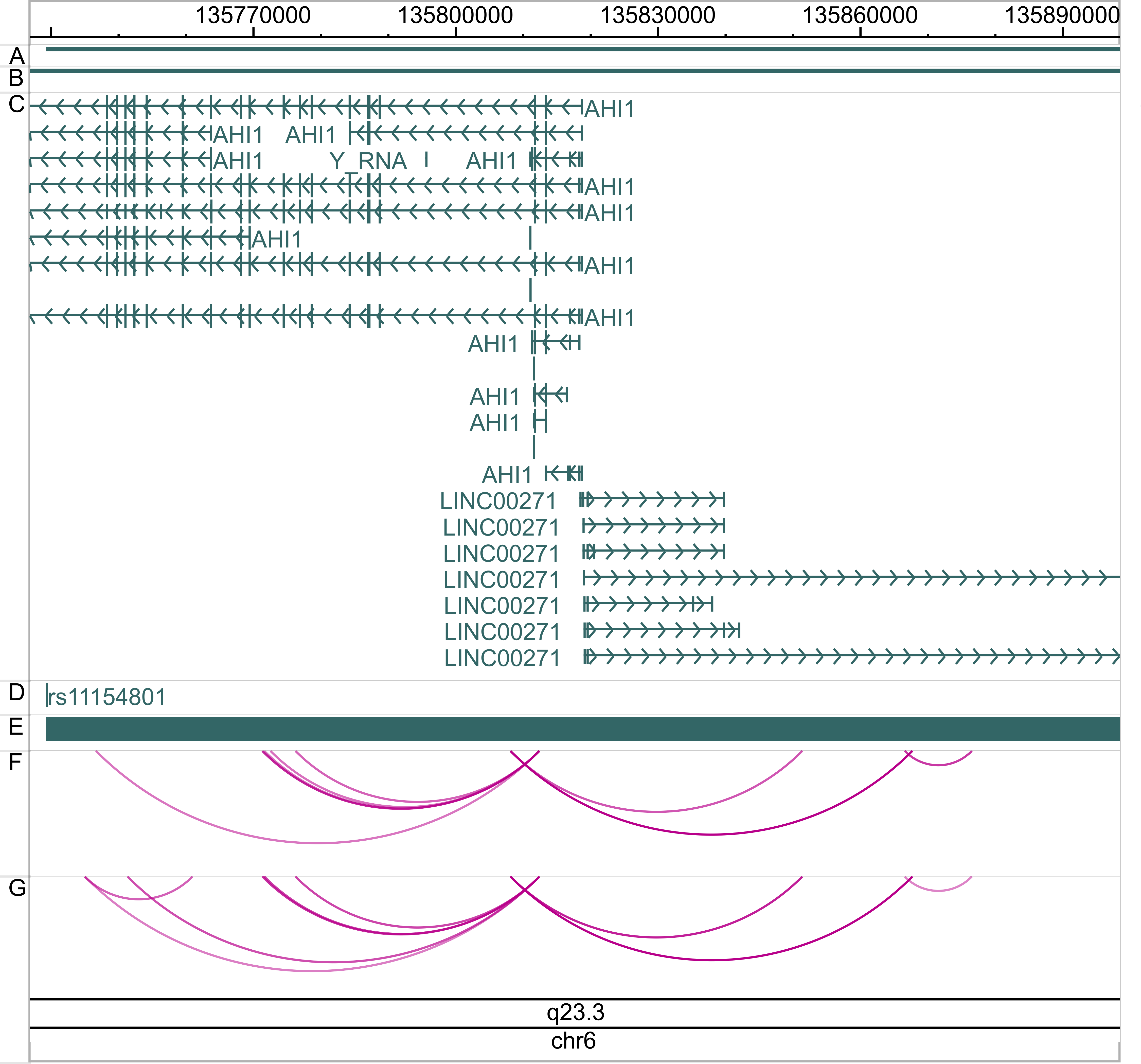

Supplement: S2 Fig — Tracks are labelled as follows: A–LD regions targeted in ‘region’ Capture Hi-C; B–Gene regions targeted in ‘promoter’ Capture Hi-C; C–RefSeq genes (packed for clarity); D–MS index SNPs; E–MS LD regions; F–Interactions observed in the GM12878 B-cell line and G–Interactions observed in the Jurkat T-cell line. All co-ordinates are based on GRCh37. (TIF) [file pone.0166923.s005.tif]
